# Clinical application prospects of traditional Chinese medicine as adjuvant therapy for metabolic reprogramming in colorectal cancer

**DOI:** 10.3389/fimmu.2025.1630279

**Published:** 2025-07-15

**Authors:** Liu Zhexian, Guo Xingqi, Dong Xinxin, Xia Tong, Ma Siping, Li Yanxi

**Affiliations:** Department of Colorectal Surgery, Cancer Hospital of China Medical University, Liaoning Cancer Hospital & Institute, Shenyang, Liaoning, China

**Keywords:** metabolic reprogramming, colorectal cancer, clinical applications, regulatory therapy, traditional Chinese medicine, complementary treatment

## Abstract

Colorectal cancer (CRC) is the second leading cause of cancer-related deaths globally and the thi^rd^ most commonly diagnosed malignancy, posing a major threat to public health. Clinical manifestations such as altered bowel habits (e.g., constipation, diarrhea, or pencil-thin stools), rectal bleeding, and abdominal pain or bloating may indicate CRC. A hallmark of CRC is metabolic reprogramming, which enables tumor cells to meet the bioenergetic and biosynthetic demands of rapid proliferation and survival. This reprogramming encompasses dysregulated glycolysis, amino acid metabolism, and lipid metabolism, collectively driving tumor growth, invasion, angiogenesis, and therapeutic resistance. Targeting metabolic reprogramming has emerged as a promising strategy in CRC therapy. Inhibitors of key metabolic enzymes and signaling pathways involved in glycolysis have demonstrated efficacy in preclinical and early clinical studies. Additionally, Traditional Chinese Medicine (TCM) has attracted increasing interest for its potential to modulate tumor metabolism. This review examines current evidence on marketed drugs, TCM, and the underlying metabolic mechanisms implicated in CRC treatment. While TCM shows promise as a complementary therapeutic approach, further research is essential to validate its clinical utility and mechanistic underpinnings.

## 1 Introduction

Colorectal cancer (CRC) remains a major global health challenge, ranking among the most commonly diagnosed malignancies and leading causes of cancer-related mortality worldwide (1, 2). In 2020, Asia accounted for over half of global CRC cases, with 51.8% of incidence and 52.4% of deaths occurring in the region, as reported by Global Cancer Statistics 2020 (3, 4). CRC is typically diagnosed at advanced stages due to the absence of symptoms in its early phases. The prognosis for stage IV CRC is particularly poor, with a 5-year survival rate of approximately 14% (5). Consequently, elucidating the mechanisms underlying CRC progression and drug resistance is crucial for improving therapeutic outcomes.

Metabolic reprogramming is a hallmark of CRC, enabling cancer cells to meet elevated bioenergetic and biosynthetic demands required for rapid proliferation, survival, and metastasis (6). This reprogramming encompasses multiple metabolic pathways—including those involved in glucose, lipid, and amino acid metabolism—which collectively support tumor growth, invasion, angiogenesis, and resistance to therapy (7–9). Key genetic mutations frequently observed in CRC, such as those in APC, KRAS, TP53, MYC, and SMAD4, have been shown to drive global metabolic alterations by modulating the expression and activity of critical metabolic enzymes (10–12). These insights provide valuable opportunities to curb metastasis and recurrence, thereby enhancing patient survival and quality of life.

**Figure 1:**
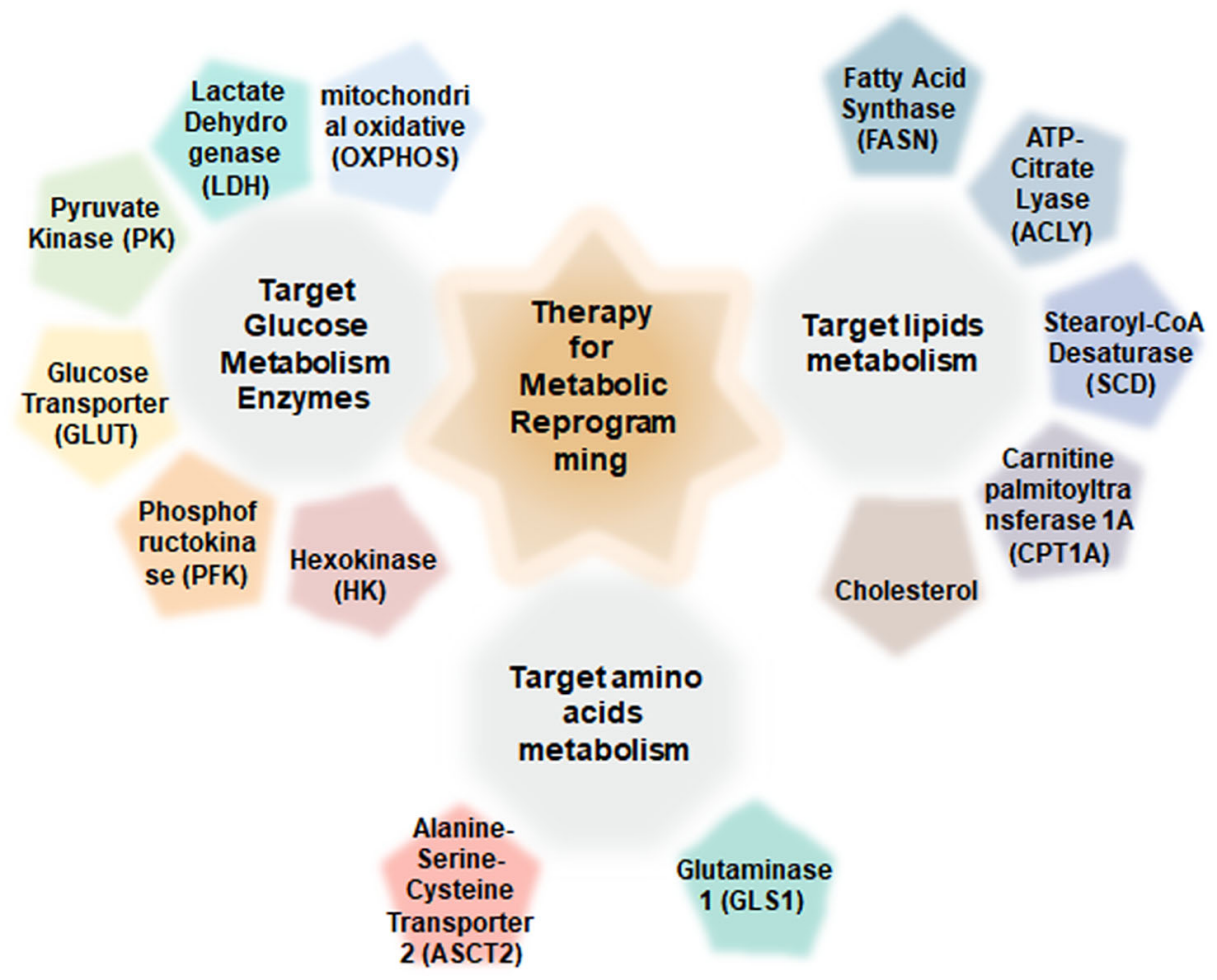
Regulatory therapy target for metabolic reprogramming in colorectal cancer.

The current therapeutic landscape for CRC includes conventional chemotherapies, targeted agents, and immunotherapies (13). A deeper understanding of the molecular mechanisms underlying metabolic dysregulation in CRC may reveal novel therapeutic targets and foster the development of more effective treatments (14). Inhibitors targeting key metabolic enzymes are emerging as promising anticancer agents (15) (**Figure 1**).

Traditional Chinese Medicine (TCM) offers compelling potential for CRC management, with several advantages over conventional therapies. Numerous chemotherapeutic agents derived from botanical sources—such as vinca alkaloids, taxanes, and camptothecins—exert their anticancer effects through disruption of metabolic pathways (16, 17). However, current research on TCM is predominantly conducted in China and often lacks diversity in cell line models, underscoring the need for broader, more inclusive studies (18). TCM not only suppresses tumor growth but also enhances the efficacy of standard chemotherapeutics by modulating cancer cell metabolism. Furthermore, due to its multi-targeted (polypharmacological) properties, TCM may alleviate cancer- and chemotherapy-induced symptoms and improve patient quality of life. Although TCM holds promise as an adjunct and chemosensitizer in CRC treatment, comprehensive research is essential to fully realize its clinical potential.

This review explores the roles of chemical and plant-derived drugs in targeting metabolic reprogramming in CRC, emphasizing their unique mechanisms of action, and outlining current challenges and future directions. A systematic search of electronic databases—including PubMed (Medline) and China National Knowledge Infrastructure (CNKI)—was conducted using predefined keywords such as “Traditional Chinese Medicine,” “Regulatory drugs,” “Colorectal cancer,” “Chemotherapeutic,” and “Metabolic reprogramming.” A total of 539 English-language publications were identified and synthesized. This study aims to advance the therapeutic application of plant-derived and regulatory drugs in CRC by elucidating their mechanisms of metabolic regulation and highlighting their potential to target critical metabolic pathways.

## 2 Conventional drugs and natural compounds that target glucose metabolism enzymes

Glucose is a primary energy source and biosynthetic substrate for cancer cells. Reprogrammed glucose metabolism ensures a continuous supply of ATP and metabolic intermediates required for the synthesis of essential macromolecules—including lipids, amino acids, and nucleic acids—thus supporting rapid tumor cell proliferation (19). Simultaneously, lactate, a glycolytic byproduct, interacts with the tumor microenvironment to facilitate immune evasion and promote tumor progression (20). Mechanistically, key oncogenes and their associated enzymes—such as hexokinase (HK), phosphofructokinase (PFK), lactate dehydrogenase A (LDHA), and pyruvate kinase isoforms M1/2 (PKM1/2) (21)—play central roles in enhancing glycolysis, thereby driving tumorigenesis, progression, and metastasis (22). Conversely, inhibition of glycolysis has been shown to suppress tumor growth in various cancers (23). Recent studies have identified several antineoplastic agents that modulate glucose metabolism by targeting key metabolic enzymes and proteins, as summarized in **Table 1**.

**Table 1:** Conventional drugs and natural compounds that target glucose metabolism enzymes.

Source	Target	Drug name	Mechanism of action	Research progress	Ref
N/A	Hexokinase (HK)	3-Bromopyruvate (3-BrPA)	Inhibits HK2 activity, suppresses glycolysis	Preclinical studies	(24)
N/A	Hexokinase (HK)	Lonidamine (LN)	Inhibits mitochondrial complexes I and II	Clinical trials	(25)
N/A	Hexokinase (HK)	2-Deoxy-D-Glucose (2-DG)	Disrupts glycolysis, reduces ATP production	Monotherapy and combination therapy in CRC	(26)
N/A	Hexokinase (HK)	Metformin	Activates AMPK, inhibits mTOR pathway	Preclinical and clinical studies	(27)
Curcuma longa	Hexokinase (HK)	Curcumin	Inhibits HK2 expression, leading to mitochondrial dysfunction and release of cytochrome C, which activates caspases	CRC cell lines	(28–30)
Green tea	Hexokinase (HK)	Epigallocatechin gallate (EGCG)	Disrupts the binding of HK2 to mitochondria, causing mitochondrial dysfunction and inducing apoptosis	CRC cell lines	(31)
Realgar	Hexokinase (HK)	Arsenic trioxide (As2O3)	Targets glucose metabolism, downregulates HK2 expression, disrupts glycolysis, and induces apoptosis	Preclinical studies	(32, 33)
N/A	Phosphofructokinase (PFK)	3PO	Inhibits PFKFB3, reduces F-2,6-BP production	Preclinical studies	(34)
N/A	Phosphofructokinase (PFK)	PFK15, PFK158	Inhibits PFKFB3, reduces glycolysis	CRC models	(35)
Oleaceae family of plants	Phosphofructokinase (PFK)	Oleanolic acid (OA)	Downregulates HIF-1α, HK2, and PFK1 expression, reducing glucose absorption and utilization, inhibiting aerobic glycolysis	CRC cell lines	(36, 37)
N/A	Glucose Transporter (GLUT)	STF-31	Inhibits GLUT1, induces apoptosis in CRC cells	Preclinical studies	(38)
N/A	Glucose Transporter (GLUT)	WZB117	Inhibits GLUT, enhances chemotherapy sensitivity	Preclinical studies	(39)
N/A	Glucose Transporter (GLUT)	BAY-876	Effectively suppresses GLUT1, with favorable metabolic stability in vitro and high oral bioavailability in vivo	Preclinical studies	(40, 41)
N/A	Glucose Transporter (GLUT)	CG-5	Inhibits GLUT, obstructs glucose transport in T cells, impedes glycolysis	Preclinical studies	(42)
*Helminthosporium dematium*	Glucose Transporter (GLUT)	Cytochalasin B	Inhibits GLUT1, decreasing intracellular glucose levels, limiting glycolysis and oxidative phosphorylation	CRC cell lines	(43, 44)
Malus domestica	Glucose Transporter (GLUT)	Phloretin	Blocks transcription factor HNF6, downregulates GLUT2 mRNA and protein, stimulates p53 pathway	CRC cell lines	(45)
Fruits, vegetables, herbs (e.g., parsley, celery, onions, chamomile)	Glucose Transporter (GLUT)	Apigenin	Downregulates GLUT1 expression (partially via HIF-1α inhibition), decreases VEGF release	CRC cell lines	(46–48)
Non-reducing disaccharide	Glucose Transporter (GLUT)	Trehalose	Inhibits cellular import of GLUT transporters, activates autophagy via AMPK-dependent pathways	CRC cell lines	(49, 50)
Milk thistle plant (Silybum marianum)	Glucose Transporter (GLUT)	Silibinin	Induces oxidative stress, inhibits PI3K-Akt-mTOR pathway, activates ERK1/2 pathway	CRC cell lines	(51, 52)
Soybeans and other legumes	Glucose Transporter (GLUT)	Genistein	Downregulates HIF-1α, inactivates GLUT1 and HK2	CRC cell lines	(53–55)
N/A	Pyruvate Kinase (PK)	Metformin	Inhibits PKM2, reduces glycolysis	CRC models	(56)
N/A	Pyruvate Kinase (PK)	Vitamin K (VK)	Inhibits PKM2, enhances chemotherapeutic effects	Preclinical studies	(57)
N/A	Pyruvate Kinase (PK)	shikonin	Inhibit PKM2 without affecting the PKM1 isoform	CRC cell lines	(58)
bark of the Tabebuia avellanedae tree	Pyruvate Kinase (PK)	lapachol	Inhibits PKM2 and reduced ATP levels	CRC cell lines	(59)
Myrtaceae family plants	Pyruvate Kinase (PK)	Clove	Reduced glucose uptake, lactate production, pyruvate kinase activity, and pyruvate production	CRC cell lines	(60)
N/A	Lactate Dehydrogenase (LDH)	FX11	Inhibits LDHA, reduces lactate production	Preclinical studies	(61)
Gossypium spp.	Lactate Dehydrogenase (LDH)	Gossypol	Inhibits LDHA, induces apoptosis	CRC models	(62)
N/A	Lactate Dehydrogenase (LDH)	Galloflavin	Binds to the NADH-binding site of LDHA, preventing its binding to single-stranded DNA, modulates inflammatory microenvironment by targeting NLRP3	CRC models	(63, 64)
Oxalic acid	Lactate Dehydrogenase (LDH)	Oxamate	Disrupts lactate production, induces mitochondrial apoptosis in CRC cells	Preclinical studies	(65)
N/A	mitochondrial oxidative phosphorylation (OXPHOS)	Metformin	Inhibits complex I of the ETC, generates ROS	CRC cell lines	(66)
N/A	mitochondrial oxidative phosphorylation (OXPHOS)	Tamoxifen	Targets mitochondria, inhibits complex I, increases ROS	CRC cell lines	(67)
N/A	mitochondrial oxidative phosphorylation (OXPHOS)	MitoTam	Inhibits complex I, increases ROS, induces cell death	CRC cell lines	(68)
N/A	mitochondrial oxidative phosphorylation (OXPHOS)	α-Tocopheryl succinate (α-TOS)	Impairs ETC activity, generates ROS	CRC cell lines	(66)
N/A	mitochondrial oxidative phosphorylation (OXPHOS)	3-BrPA	Inhibits glycolysis and OXPHOS	CRC cell lines	(66)
N/A	mitochondrial oxidative phosphorylation (OXPHOS)	ME-344	Targets complex I, induces cell death via mitochondrial permeability transition	CRC cell lines	(69)
N/A	mitochondrial oxidative phosphorylation (OXPHOS)	ME-143	Inhibits complex I, obstructs electron flow	CRC cell lines	(70)
Rheum palmatum	mitochondrial oxidative phosphorylation (OXPHOS)	Rhein-DCA	Targets mitochondria, inhibits glycolysis via PDK-PDH axis, disrupts OXPHOS	CRC cell lines	(71)
Marine organisms	mitochondrial oxidative phosphorylation (OXPHOS)	Frondoside A	Induces apoptosis via mitochondrial pathway, increases ROS, releases cytochrome c	CRC cell lines	(72)
Ginseng	mitochondrial oxidative phosphorylation (OXPHOS)	Ginsenoside compound K	Induces ROS-mediated apoptosis via mitochondrial pathway, activates caspase-9 and caspase-3	CRC cell lines	(73)

### 2.1 Targeting hexokinase

Hexokinase (HK), the first rate-limiting enzyme in glycolysis, catalyzes the conversion of glucose to glucose-6-phosphate [G-6-P (23, 74)], a pivotal intermediate in glycolysis, the pentose phosphate pathway (PPP), and glycogen synthesis. Among glycolytic enzymes, HK is considered the most critical regulatory node in glucose metabolism. Mammalian cells express four HK isoforms: HK1, HK2, HK3, and HK4 (24).

Several HK inhibitors have demonstrated anticancer activity, with the most extensively studied being 3-bromopyruvate (3-BrPA), lonidamine (LN), 2-deoxy-D-glucose (2-DG), and metformin. 3-BrPA directly inhibits HK2, thereby suppressing glycolytic flux in cancer cells (75). In addition to its glycolytic inhibition, 3-BrPA enhances the cytotoxicity of chemotherapeutic agents and mitigates multidrug resistance (MDR), a major mechanism of therapeutic failure in cancer by promoting drug efflux (25, 76). Lonidamine (LN), an adenine nucleotide translocator (ANT) ligand, inhibits mitochondrial complexes I and II and promotes the formation of mitochondrial permeability transition pores (77, 78). It is a novel glycolysis-targeting agent currently undergoing clinical evaluation for the treatment of various cancers, including ovarian, breast, and lung malignancies (26). 2-Deoxy-D-glucose (2-DG), a glucose analog, interferes with glycolysis and ATP synthesis, leading to energy depletion and cancer cell death. In CRC, 2-DG has shown antitumor efficacy both as monotherapy and in combination with chemotherapy and radiotherapy (26, 27). Metformin, another HK2 inhibitor, exerts its effects primarily by activating AMP-activated protein kinase (AMPK), which subsequently suppresses the mTOR pathway, thereby reducing glycolysis and cell proliferation (79). In CRC models, metformin decreases glucose uptake and lactate production, ultimately inhibiting tumor growth and enhancing chemosensitivity (28).

Several natural compounds also inhibit HK2 and exert pro-apoptotic effects in cancer cells. Curcumin, a polyphenolic compound derived from *Curcuma longa*, has been widely investigated for its anticancer potential (29, 30). By downregulating HK2, curcumin induces mitochondrial dysfunction and the release of cytochrome c, activating caspases and promoting apoptosis in CRC cells (31). Epigallocatechin gallate (EGCG), a major polyphenol in green tea, inhibits the anchorage-independent growth of CRC cells by disrupting the interaction between HK2 and mitochondria. This mitochondrial disruption impairs energy metabolism and induces apoptosis (32). Arsenic trioxide (As_2_O_3_), an established therapeutic agent for acute promyelocytic leukemia (APL) (33), has also been shown to inhibit HK2 expression and glycolysis in cancer cells. By downregulating glucose metabolism and inducing apoptosis, As_2_O_3_ offers a unique mechanism for suppressing tumor growth (80).

### 2.2 Targeting phosphofructokinase

PFK, the second major rate-limiting enzyme in glycolysis, catalyzes the conversion of fructose-6-phosphate (F-6-P) to fructose-1,6-bisphosphate (F-1,6-BP) (81). PFKFB3 (6-phosphofructo-2-kinase/fructose-2,6-bisphosphatase 3) is a critical glycolytic regulator that promotes the synthesis of fructose-2,6-bisphosphate (F-2,6-BP), a potent allosteric activator of PFK1. PFKFB3 is frequently overexpressed in various cancers and is associated with lymph node metastasis and poor survival outcomes (34).

Several PFKFB3 inhibitors—including 3PO, PFK15, and PFK158—have been identified as potential therapeutic agents. Administration of 3PO leads to a rapid reduction in glucose uptake, lactate production, and ATP synthesis. Additionally, 3PO can reprogram the metabolic profile of patient-derived tumor organoids to favor oxidative phosphorylation. *In vivo*, neoadjuvant treatment with 3PO promotes vascular normalization, alleviates hypoxia, and enhances tumor necrosis (35). PFK15 and PFK158, derivatives of 3PO, have also demonstrated efficacy in attenuating glycolytic activity and suppressing tumor growth in colorectal cancer (CRC) models. Notably, PFK15 exhibits approximately 100-fold greater inhibitory potency against PFKFB3 compared to 3PO (82). PFK15 significantly reduces F-2,6-bisphosphate (F-2,6-BP) levels in xenograft tumors and induces apoptosis in transformed cancer cells in both *in vivo* and *in vitro* settings (82). PFK158 is currently being evaluated in a Phase I, dose-escalation, multicenter clinical trial (NCT02044861) aimed at assessing its safety, tolerability, and pharmacokinetics in patients with advanced solid tumors. Preliminary findings revealed antitumor activity in 6 of 19 evaluable patients. Although the trial did not meet the desired efficacy endpoints, it underscored the therapeutic potential of targeting PFKFB3 in cancer treatment (36, 83).

In traditional Chinese medicine (TCM), oleanolic acid (OA) has emerged as a potential therapeutic candidate for gastric cancer (37). OA, a triterpenoid compound abundant in plants of the Oleaceae family, modulates aerobic glycolysis and tumor cell proliferation. Specifically, OA inhibits gastric cancer cell growth and reduces intracellular lactate levels by suppressing glucose uptake and utilization through downregulation of HIF-1α, HK2, and PFK1 expression (84).

### 2.3 Targeting glucose transporter

Glucose transporters (GLUTs) are integral membrane proteins responsible for facilitating glucose entry into cells. Among the best-characterized subtypes are GLUT1, GLUT2 (SLC2A2), GLUT3 (SLC2A3), and GLUT4 (SLC2A4), each exhibiting distinct regulatory mechanisms and kinetic properties, thereby playing specialized roles in maintaining cellular and systemic glucose homeostasis (85, 86). GLUT1 is implicated in chemoresistance via its regulation of glycolysis (87), while elevated expression of GLUT2 and GLUT3 correlates with poor prognosis in CRC (38). Consequently, GLUTs represent attractive therapeutic targets for disrupting glucose metabolism in CRC.

Several small-molecule inhibitors targeting GLUTs have shown preclinical promise, including STF-31, WZB117, BAY-876, and CG-5. STF-31 selectively induces apoptosis in cancer cells without affecting normal tissues, thereby reducing CRC cell viability and proliferation (39). WZB117 triggers CRC cell death, particularly when delivered via hypoxia-responsive nanoparticles (88), and has also been shown to resensitize 5-fluorouracil (5-FU)-resistant colon cancer cells to chemotherapeutic agents, supporting its potential as an adjuvant for overcoming drug resistance (40). BAY-876 is a potent and selective GLUT1 inhibitor with high metabolic stability *in vitro* and favorable oral bioavailability *in vivo*; it has demonstrated antitumor efficacy in several cancers, including ovarian and triple-negative breast cancer (41, 42). CG-5, a thiazolidinedione derivative, inhibits GLUT-mediated glucose transport in T cells, disrupts glycolysis, and impairs Th1 and Th17 cell differentiation while promoting Treg cell development and reducing CD4+ T cell proliferation (43).

Natural compounds have emerged as promising GLUT inhibitors with potential anticancer effects in CRC. Key examples include cytochalasin B, phloretin, apigenin, trehalose, silibinin, and genistein. Cytochalasin B, the first identified GLUT1 inhibitor, has provided critical insights into CRC metabolism (44). By inhibiting GLUT1, it reduces intracellular glucose availability, thereby limiting substrates for glycolysis and oxidative phosphorylation (45). Phloretin, a natural GLUT2 inhibitor, suppresses GLUT2 expression by blocking the transcription factor HNF6. It also activates the p53 pathway, promoting cell cycle arrest and apoptosis—mechanisms that collectively inhibit tumor progression and enhance CRC cell sensitivity to other therapies (46). Apigenin, a flavonoid found in parsley, celery, onions, and chamomile (47), inhibits CRC cell proliferation and induces apoptosis through downregulation of GLUT1, partially via HIF-1α inhibition (48). Furthermore, Apigenin reduces VEGF secretion under both normoxic and hypoxic conditions, highlighting its anti-metastatic potential (49). Trehalose, a non-reducing disaccharide composed of two glucose units linked via an α,α-1,1-glycosidic bond (50), impedes glucose transporter activity and induces a starvation-like state characterized by ATP depletion. This metabolic stress activates autophagy through AMPK-dependent signaling, contributing to its anticancer effects (51). Silibinin, a flavonoid derived from *Silybum marianum* (52) (milk thistle), rapidly induces oxidative stress in CRC cells. It disrupts energy homeostasis by inhibiting the PI3K-Akt-mTOR pathway and activating ERK1/2, leading to metabolic reprogramming (53). Genistein, an isoflavone abundant in soybeans and legumes (54), suppresses GLUT1 and HK2 by downregulating HIF-1α (55). It also induces cell cycle arrest and reduces invasion capacity in CRC cells (54).

### 2.4 Targeting pyruvate kinase

The third rate-limiting step in glycolysis is catalyzed by pyruvate kinase (PK), which converts phosphoenolpyruvate to (89)pyruvate. In mammals, PK exists in four isoforms: PKM1, PKM2, PKR, and PKL (56), among which PKM2 is predominant in cancer cells. Inhibition of PKM2 disrupts glycolysis and promotes apoptosis.

Three principal classes of PKM2 inhibitors have been identified: metformin, vitamin K, and shikonin. Metformin suppresses PKM2 expression and inhibits tumor growth (90) by modulating AMPK and mTOR signaling. In CRC xenograft models, metformin significantly reduces tumor volume through these pathways (57). Vitamin K (VK), a lipophilic naphthoquinone, exhibits isoform-specific inhibition, with VK3 and VK5 more potently targeting PKM2 over PKM1 (91). The combination of VK3 and vitamin C has shown enhanced anticancer efficacy, and clinical studies suggest VK3 can overcome resistance to chemotherapeutics such as doxorubicin and adriamycin (92). VK2 has also been reported to inhibit CRC cell proliferation by suppressing NF-κB signaling and inducing pro-apoptotic proteins (58).

Shikonin, a naturally occurring compound, selectively inhibits PKM2 without affecting PKM1. It reduces glucose uptake and lactate production, underscoring its therapeutic promise in cancer metabolism (59). Similarly, lapachol, a naphthoquinone derived from the bark of *Tabebuia avellanedae*, inhibits PKM2 activity and reduces ATP levels (60). An active fraction from clove (*Eugenia caryophyllata* or *Syzygium aromaticum*), referred to as AFC, has been shown to decrease glucose uptake, lactate production, PK activity, and pyruvate synthesis in CRC cells via PKM2 downregulation, ultimately attenuating aerobic glycolysis (93).

### 2.5 Targeting lactate dehydrogenase

Lactate dehydrogenase (LDH) catalyzes the final step of glycolysis by reversibly converting pyruvate to lactate. The human genome encodes four LDH isoforms—LDHA, LDHB, LDHC, and LDHD (61). Among these, LDHA and LDHB are highly expressed in malignancies, with LDHA predominantly converting pyruvate to lactate and LDHB catalyzing the reverse reaction. Elevated LDHA expression is associated with poor prognosis across multiple tumor types.

FX11, a small-molecule LDHA inhibitor, binds directly to its active site, blocking pyruvate-to-lactate conversion and thereby reducing cancer cell invasiveness and metastatic potential (94). In preclinical xenograft models of human lymphoma and pancreatic cancer, FX11 exhibited significant antitumor activity (95).

The natural compound gossypol, a nonselective LDHA inhibitor, has demonstrated potent anticancer effects *in vitro* and in animal models (62). In CRC cells, gossypol suppresses LDHA activity, lowering lactate production and glycolytic flux. This metabolic disruption induces bioenergetic and oxidative stress, resulting in cell cycle arrest and apoptosis (96). In CRC xenograft models, gossypol markedly inhibited tumor growth and progression (63). Galloflavin, another LDHA inhibitor, binds to the enzyme’s NADH-binding site, blocking its activity and impeding CRC proliferation (64). Additionally, Galloflavin modulates the tumor inflammatory microenvironment by targeting NLRP3 and downregulating oncogenic c-Myc and P21, further enhancing its antitumor efficacy (65). Oxamate, an LDHA inhibitor, induces mitochondrial apoptosis in CRC cells by suppressing lactate synthesis. In combination with metformin or mTOR inhibitors, it shows synergistic antitumor effects in preclinical models (97).

### 2.6 Targeting mitochondrial oxidative phosphorylation

To meet increased energy and biosynthetic demands, cancer cells often augment oxidative phosphorylation (OXPHOS) (98).Inhibiting OXPHOS suppresses proliferation and tumorigenicity even in glycolysis-competent CRC cells, both *in vitro* and in patient-derived xenografts (66). Proper electron transport chain (ETC) function is essential for OXPHOS and ATP production, both critical for carcinogenesis. ETC inhibitors—such as metformin, tamoxifen, α-tocopheryl succinate (α-TOS), 3-bromopyruvate (3-BrPA), and ME-series inhibitors—disrupt respiratory complex activity, increase reactive oxygen species (ROS) levels, and trigger apoptosis (67). Tamoxifen, traditionally used as a selective estrogen receptor modulator in breast cancer, also targets mitochondria (68). Its derivative, MitoTam, localizes to mitochondria, inhibits complex I, elevates ROS, and induces cell death in breast cancer cells (69). The small-molecule inhibitor ME-344 effectively inhibits complex I and multiple pro-death signaling pathways associated with mitochondrial permeability transition in CRC (70). Both ME-143 and ME-344 disrupt NADH oxidation at complex I, blocking electron flow through the ETC. ME-344 further induces Bax translocation to the mitochondrial outer membrane, triggering mitochondrial permeability transition and releasing pro-apoptotic molecules (71).

The natural product Rhein, known for its mitochondrial-targeting properties, has been conjugated with dichloroacetate (DCA) to create Rhein-DCA, a dual glycolysis and OXPHOS inhibitor. Rhein-DCA accumulates in mitochondria, inhibits glycolysis via the PDK-PDH axis, and disrupts the respiratory chain. In CRC models, it induces oxidative stress, decreases lactate levels, and promotes immunogenic cell death (72). Frondoside A, a triterpene glycoside derived from marine organisms, induces mitochondrial apoptosis by decreasing antiapoptotic proteins Bcl-2 and survivin, increasing ROS production, and promoting cytochrome c release (73). Similarly, ginsenoside compound K, a natural derivative of ginseng, activates ROS-mediated mitochondrial apoptosis, leading to cytochrome c release and caspase-9/-3 activation (99). These natural compounds offer promising avenues for CRC therapy by targeting mitochondrial function and enhancing oxidative stress-induced cell death.

## 3 Conventional drugs and natural compounds that target lipid metabolism

Modulating key regulators of lipid metabolism represents a promising strategy to counteract metabolic reprogramming in malignancies, underscoring the urgent need for novel therapeutic targets to improve cancer treatment and prognosis (100, 101). Although no lipid-targeting therapeutics are currently approved for CRC, numerous small-molecule inhibitors that interfere with lipid metabolism have shown preclinical efficacy and may enhance therapeutic outcomes when used in combination regimens (102)(**Table 2**).

**Table 2:** Conventional drugs and natural compounds that target lipids metabolism.

Source	Target	Drug name	Mechanism of action	Research progress	Ref
N/A	Fatty Acid Synthase (FASN)	Cerulenin	Inhibits FASN, induces apoptosis	CRC cell lines	(103)
N/A	Fatty Acid Synthase (FASN)	C75 , C93	direct or indirect effects on CPT1 to enhance fatty acid oxidation	CRC models	(104)
N/A	Fatty Acid Synthase (FASN)	Orlistat	Activates caspase-3, reduces fatty acid synthesis	CRC cell lines	(105)
N/A	Fatty Acid Synthase (FASN)	TVB-3664, TVB-3166, TVB-2640	antitumor efficacy in both in vivo and in vitro studies and are now undergoing clinical trials in colorectal cancer patients	CRC models	(106)
Chrysanthemum morifolium	Fatty Acid Synthase (FASN)	Luteolin	Inhibits cell cycle, downregulates anti-apoptotic proteins	CRC cell lines	(107)
Vitis vinifera	Fatty Acid Synthase (FASN)	Resveratrol	Suppresses proliferation, decreases abnormal crypt foci development;Promotes apoptosis, suppresses cell proliferation by modulating growth factor 1 receptor/AKT/Wnt signaling pathway and activating p53	CRC cell lines	(108, 109)
N/A	ATP-Citrate Lyase (ACLY)	ETC-1002	Inhibits ACLY, reduces fatty acid synthesis	Clinical trials	(110)
Curcuma longa	ATP-Citrate Lyase (ACLY)	Curcumin	reducing acetyl-CoA levels and disrupting lipid synthesis	CRC cell lines	(111)
Coptis chinensis	ATP-Citrate Lyase (ACLY)	berberine	modulate lipid metabolism by inhibiting ACLY	CRC models	(112)
N/A	Stearoyl-CoA Desaturase (SCD)	T-3764518	Inhibits SCD1, induces apoptosis	CRC models	(113)
Betula platyphylla	Stearoyl-CoA Desaturase (SCD)	Betulinic Acid	Inhibits SCD1, arrests cell cycle	CRC cell lines	(114)
N/A	Carnitine palmitoyltransferase 1A (CPT1A)	Etomoxir	Markedly reduces fatty acid absorption and ATP generation	CRC cell lines	(115, 116)
N/A	Carnitine palmitoyltransferase 1A (CPT2A)	Perhexiline	Increases ROS production and apoptosis	CRC cell lines	(117, 118)
N/A	Cholesterol	Lovastatin	Inhibits cholesterol synthesis, inhibits Wnt signaling pathway	CRC cell lines	(119)
Citrus sinensis	Cholesterol	hesperetin	Decreases foam cell formation, intracellular cholesterol concentrations	CRC cell lines	(120)

### 3.1 Targeting fatty acid synthase

Fatty acid synthase (FASN), a pivotal enzyme in *de novo* lipogenesis, is inversely correlated with CRC prognosis (102, 121). Several FASN-specific inhibitors—such as cerulenin, C75, Orlistat, and TVB-series compounds—have demonstrated pro-apoptotic activity and therapeutic potential across various cancer types (103).

Cerulenin induces apoptosis in CRC cell lines by activating the caspase cascade and inhibiting DNA replication and S-phase progression (122). In HT-29 and LoVo cells, cerulenin also disrupts energy metabolism and inhibits mTOR signaling, thereby suppressing the malignant phenotype of CRC (123). Combination therapy with cerulenin and oxaliplatin may attenuate oxaliplatin-induced neurotoxicity, reduce required dosages, and improve long-term chemotherapeutic tolerance in clinical trials (124). C75 and C93 structurally related to cerulenin, also target FASN (105). Both compounds, including cerulenin, influence carnitine palmitoyltransferase 1 (CPT1), thereby enhancing fatty acid oxidation (104, 105). Notably, treatment with C75 and cerulenin significantly reduces food intake and body weight in murine models (105). Orlistat, another FASN inhibitor, activates caspase-3 in a dose-dependent manner, induces G1 cell cycle arrest, and reduces both proliferation and lipid synthesis in HT-29 cells (106). Second-generation FASN inhibitors—TVB-3664, TVB-3166, and TVB-2640—exhibit potent antitumor activity *in vitro* and *in vivo* and are currently undergoing clinical evaluation in CRC patients (107).

In addition to synthetic compounds, natural products such as luteolin and resveratrol also exhibit FASN-inhibitory and anticancer properties. In HT-29 cells, luteolin downregulates anti-apoptotic proteins and induces cell cycle arrest (108). Resveratrol inhibits proliferation in Caco-2 cells, suppresses aberrant crypt foci formation (109), and induces apoptosis by modulating the IGF1R/AKT/Wnt pathway and activating p53 (125).

### 3.2 Targeting ATP‐citrate lyase

ATP citrate lyase (ACLY), which catalyzes the conversion of citrate to acetyl-CoA, acts as a rate-limiting enzyme in early lipid biosynthesis. ACLY has been implicated in promoting CRC progression in both *in vitro* and *in vivo* models (110). ETC-1002, a potent ACLY inhibitor, activates the AMPK pathway and suppresses lipid and cholesterol synthesis, although its clinical efficacy has been predominantly observed in cholesterol regulation (126). Nonetheless, co-administration of ETC-1002 with the IGF1R inhibitor linsitinib has demonstrated significant synergistic effects in inhibiting CRC metastasis (111). Curcumin, a natural compound, also inhibits ACLY activity, lowering acetyl-CoA levels and disrupting lipid synthesis essential for tumor growth (112). Other natural agents, such as berberine, similarly suppress ACLY activity, thereby attenuating tumor progression and metastatic potential (127).

### 3.3 Targeting stearoyl‐CoA desaturase

Stearoyl-CoA desaturase (SCD), an endoplasmic reticulum membrane enzyme, catalyzes the conversion of saturated fatty acids to monounsaturated fatty acids, thus facilitating lipid biosynthesis (128). Elevated expression of SCD1, the predominant isoform, is negatively associated with CRC prognosis (113). The novel oral SCD inhibitor T-3764518 has been shown to promote apoptosis by disrupting lipid raft integrity and inhibiting oncogenic signaling in CRC xenograft models (114). Betulinic acid, a natural SCD1 inhibitor derived from birch bark, induces G2/M cell cycle arrest and inhibits CRC growth (129). Furthermore, it reduces clonogenicity and induces apoptosis in CRC stem-like cells, highlighting its potential as a therapeutic agent targeting cancer stemness (130).

### 3.4 Targeting carnitine palmitoyltransferase 1A

Carnitine palmitoyltransferase 1A (CPT1A) is a key rate-limiting enzyme in fatty acid oxidation. CPT1A-mediated β-oxidation supports reactive oxygen species (ROS) detoxification and enhances reduced glutathione synthesis by increasing intracellular NADPH levels (115). Etomoxir, an irreversible CPT1 inhibitor, significantly impairs fatty acid uptake and ATP production without affecting tumor cell stemness or angiogenesis (115, 116). Notably, combining etomoxir with cisplatin enhances cisplatin-induced apoptosis in HCT116 colorectal cancer (CRC) cells in a dose-dependent manner (117). Another CPT1 inhibitor, perhexiline, induces ROS accumulation and apoptosis, thereby suppressing gastrointestinal tumor progression (118). Co-treatment with perhexiline and oxaliplatin further promotes apoptosis and sensitizes HCT116 cells to oxaliplatin (131).

### 3.5 Targeting cholesterol

Numerous studies have established a positive correlation between elevated dietary and plasma cholesterol levels and increased CRC risk, whereas statin-mediated inhibition of cholesterol biosynthesis is associated with reduced risk (119, 132). Lovastatin inhibits both canonical Wnt signaling and alternative oncogenic pathways, such as YAP/TAZ, thereby suppressing CRC progression (133). At low doses, lovastatin promotes CRC cell differentiation and significantly increases their sensitivity to 5-fluorouracil (5-FU) (120). Hesperetin, a cholesterol-lowering flavonoid found in citrus juices, reduces foam cell formation, intracellular cholesterol levels, and cholesterol esterification, while enhancing cholesterol efflux in THP-1 macrophages (134). These findings suggest that hesperetin may also regulate cholesterol metabolism and inhibit CRC progression.

## 4 Conventional drugs and natural compounds that target amino acid metabolism

Amino acids serve as critical metabolic intermediates linking glucose and lipid metabolism. Under glutamine-deprived conditions, CRC cells activate autophagy to maintain amino acid homeostasis and intracellular metabolic balance (135)(**Table 3**).

**Table 3:** Conventional drugs and natural compounds that target amino acids metabolism.

Source	Target	Drug name	Mechanism of action	Research progress	Ref
N/A	Glutaminase 1 (GLS1)	BPTES	Inhibits GLS1, reduces glutamine metabolism	CRC models	(136)
N/A	Glutaminase 1 (GLS1)	CB-839	Inhibits GLS1, enhances chemotherapeutic effects	Clinical trials	(137)
N/A	Glutaminase 1 (GLS1)	Compound 968	Inhibits GLS2,offering an alternate inhibitory mechanism	CRC cell lines	(138)
N/A	Alanine-Serine-Cysteine Transporter 2 (ASCT2)	Ab3-8	Inhibits ASCT2, reduces glutamine uptake	CRC models	(139)
N/A	Alanine-Serine-Cysteine Transporter 2 (ASCT2)	V-9302	Inhibits ASCT2, reduces glutamine uptake	Preclinical studies	(140)

### 4.1 Targeting glutaminase 1

Glutaminase 1 (GLS1) catalyzes the conversion of glutamine to glutamate, and its overexpression is strongly associated with poor prognosis in multiple cancers. Inhibiting GLS1 may disrupt glutamine metabolism and hinder tumor progression (136). BPTES (Bis-2-(5-phenylacetamido-1,2,4-thiadiazol-2-yl)ethyl sulfide), a potent GLS1 inhibitor, suppresses glutamine utilization and inhibits CRC growth (141), although its clinical development is limited by poor solubility and metabolic instability (137). CB-839, a selective and clinically advanced GLS1 inhibitor, has demonstrated promising results. In a phase II trial, CB-839 combined with 5-FU extended progression-free survival beyond 6 months in 21.8% of patients, potentially through modulation of neutrophil extracellular traps (142). Ongoing clinical trials are evaluating CB-839 in combination with palbociclib for KRAS-mutant CRC and with nivolumab for melanoma and renal cell carcinoma (138). Compound 968, another GLS1 inhibitor with a distinct mechanism of action, has shown broad anticancer efficacy across multiple cell lines (143).

### 4.2 Targeting alanine-serine-cysteine transporter 2

The amino acid transporter ASCT2 has emerged as a critical pro-tumorigenic factor, with elevated expression linked to poor prognosis in various cancers (139). In CRC, ASCT2 overexpression is strongly associated with KRAS mutations. Given the therapeutic resistance commonly observed in KRAS-mutant tumors, ASCT2 represents a promising target for this CRC subset (144, 145).

A monoclonal antibody against ASCT2, Ab3-8, significantly reduced glutamine uptake and inhibited AKT and ERK phosphorylation in SW1116 and HCT116 CRC cells *in vitro*. *In vivo*, Ab3–8 treatment markedly suppressed tumor growth in KRAS-mutant CRC xenografts. Additionally, V-9302, a small-molecule ASCT2 inhibitor, competitively blocks glutamine transport. Combined with ASCT2 gene silencing, V-9302 further impairs glutamine uptake and significantly inhibits tumor progression in preclinical models (140).

## 5 Current clinical status of the metabolic mechanisms in CRC

Based on the metabolic mechanisms of glucose, lipid, and amino acid pathways in CRC discussed in our previous dialogue and further supported by recent research, we present a comprehensive overview of drugs currently under clinical investigation or already approved for CRC treatment. These are categorized by metabolic targets in **Table 4**.

**Table 4:** The current clinical status of the metabolic mechanisms involved in the CRC.

Metabolic mechanism	Target	Drug(s)	Status & indication	Key findings/mechanism
Glucose Metabolism	HK2	Metformin	Approved (Type 2 diabetes); Clinical trials for CRC (chemosensitizer)	Inhibits glycolysis, enhances 5-FU efficacy; reduces tumor growth via AMPK/mTOR suppression .
Glucose Metabolism	PFKFB3	PFK158	Phase I (NCT02044861) for advanced solid tumors	Suppresses glycolytic flux; combats chemoresistance in CRC models .
Glucose Metabolism	GLUT1/3	BAY-876	Preclinical/Phase I for ovarian and CRC	Blocks glucose uptake; synergizes with immunotherapy .
Glucose Metabolism	LDHA	Gossypol (AT-101)	Phase II for CRC and other cancers	Inhibits lactate production; induces oxidative stress and apoptosis .
Lipid Metabolism	FASN	TVB-2640	Phase II for KRAS-mutant CRC (NCT03808558)	Suppresses lipogenesis; enhances efficacy of chemotherapy and targeted therapies .
Lipid Metabolism	SCD1	TVB-3664 (Kovanet)	Phase I for solid tumors	Inhibits monounsaturated fatty acid synthesis; disrupts membrane integrity in CRC .
Lipid Metabolism	CPT1A	Etomoxir	Preclinical/Phase I for CRC	Blocks fatty acid oxidation; synergizes with cisplatin and 5-FU .
Lipid Metabolism	HMG-CoA reductase	Lovastatin	Approved (hyperlipidemia); Clinical trials for CRC adjuvant therapy	Inhibits cholesterol synthesis; sensitizes CRC cells to 5-FU via Wnt/YAP suppression .
Amino Acid Metabolism	GLS1	CB-839 (Telaglenastat)	Phase II for KRAS-mutant CRC (with palbociclib)	Depletes glutamine; suppresses mTOR signaling and tumor growth .
Amino Acid Metabolism	ASCT2	V-9302	Preclinical for KRAS-mutant CRC	Competitive inhibitor of glutamine uptake; induces metabolic crisis in chemoresistant cells .
Amino Acid Metabolism	Arginine depletion	PEGylated arginase (ADI-PEG20)	Phase II for CRC (with 5-FU)	Deprives arginine; triggers ferroptosis and enhances chemotherapy response .

A systematic review of randomized controlled trials (RCTs) involving traditional Chinese medicine (TCM) in CRC identified 1,778 RCTs published from database inception through August 1, 2023 (146). The publication volume has steadily increased, reflecting growing interest and research activity in this area. However, most trials feature small sample sizes, typically ranging from 60 to 100 participants, and intervention durations commonly span 4, 8, or 12 weeks. Interventions include various TCM modalities such as decoctions, injections, patent medicines, and acupuncture.

Despite this growing body of work, several critical limitations remain in current TCM-CRC RCTs. First, the quality of randomization is generally poor. Only a minority of trials adequately report random sequence generation methods, increasing the risk of selection bias and undermining result validity. Second, blinding is rarely implemented effectively. The proportion of trials reporting blinding procedures is low and has declined in recent years, raising concerns about performance and detection biases due to participants and investigators being aware of treatment assignments (147). Third, small sample sizes limit statistical power, making many studies unable to detect clinically meaningful differences. Fourth, outcome measures are typically based on Western medical evaluation systems, which are complex and lack standardization. Moreover, TCM-specific indicators are rarely incorporated, and important aspects such as long-term efficacy, anxiety, and depression are insufficiently addressed.

To overcome these limitations, future TCM-CRC RCTs should adopt rigorous methodologies. This includes robust randomization techniques (e.g., computer-generated sequences, centralized randomization), effective blinding strategies (e.g., placebo controls, double-blind designs), and sample size determinations based on *a priori* power analyses. Multicenter collaborations may be necessary to achieve adequate recruitment. Studies should also incorporate long-term follow-up to assess the durability of TCM effects and evaluate both clinical and psychosocial outcomes, including quality of life. Furthermore, adherence to reporting standards such as the CONSORT guidelines will enhance transparency and reproducibility. Optimizing study protocols based on the shortcomings of prior research will be essential for improving trial quality.

In conclusion, although the number of TCM-CRC RCTs is increasing, substantial improvements in study design—particularly in randomization, blinding, sample size, and outcome measurement—are necessary to strengthen the reliability and credibility of evidence. Future research should aim to generate high-quality data to support the safety and efficacy of TCM in CRC prevention and treatment.

## 6 Major setbacks of TCMs in CRC management

Despite technological advancements, the application of TCM in CRC management still faces significant challenges. First, the inherent complexity of botanical mixtures in TCM complicates standardization, especially in multi-component formulations. This complexity also impedes the identification and quantification of bioactive constituents, which is essential for quality control. Second, mechanistic ambiguity remains a major obstacle; the multi-target interactions of TCM components are poorly defined, limiting our understanding of their pharmacodynamic and pharmacokinetic profiles in clinical settings. Third, regulatory barriers persist due to the absence of harmonized guidelines for the approval of botanical drugs. These factors collectively hinder the clinical translation and widespread integration of TCM in CRC treatment. Addressing these challenges will require the application of advanced analytical technologies, multi-omics approaches, and the development of cohesive regulatory frameworks. As research progresses, the mechanisms of action of TCMs and their individual components will become increasingly well-characterized. While TCM holds considerable promise as an adjunctive therapy in CRC, further investigation is needed to fully elucidate its therapeutic potential and establish its role in evidence-based oncology.

## 7 Discussion

CRC is the third most common malignancy globally, posing significant challenges due to its high morbidity and mortality. Over the past two decades, research into CRC pathogenesis has highlighted the pivotal role of somatic genetic alterations acquired during tumorigenesis. Increasing evidence also underscores the critical influence of epigenetic modifications, which alter transcriptional programs and consequently affect gene expression and cellular behavior in CRC. Among the hallmarks of cancer, metabolic reprogramming has emerged as a defining feature and a promising therapeutic target in solid tumors. In CRC, enzymes involved in altered metabolic pathways are frequently dysregulated to support tumor progression and enhance resistance to cellular stress. Targeting these metabolic enzymes has transformed the therapeutic landscape and improved clinical outcomes in various cancers. Given the prevalence of metabolic enzyme modifications, a deeper understanding of the downstream effector mechanisms involved in metabolic reprogramming is essential for the development of targeted therapies (148–150). This review explores key metabolic alterations in CRC and their associated therapeutic agents. Exploiting genetic and metabolic vulnerabilities offers novel avenues for the development of innovative diagnostic and therapeutic strategies. Although several pathway-specific inhibitors have demonstrated promising anticancer effects, most remain in preclinical stages, underscoring the urgent need for comprehensive prospective studies to validate their clinical efficacy.

Traditional chemotherapy remains a cornerstone of CRC treatment, offering notable benefits such as the ability to suppress or eliminate proliferating cancer cells. Combination regimens such as FOLFOX and FOLFIRI have significantly improved overall survival rates. However, chemotherapy is often accompanied by substantial drawbacks. Adverse effects—including nausea, fatigue, and immunosuppression—can markedly impair patients’ quality of life (151, 152). Moreover, therapeutic efficacy is often limited in advanced or metastatic CRC, and drug resistance frequently emerges over time (153). Despite these limitations, chemotherapy continues to play a vital role in CRC management, particularly when combined with targeted therapies (154).

The integration of TCMs into CRC treatment presents several challenges that hinder their broader clinical application. The inherent complexity of multi-component botanical formulations complicates standardization and quality control, as the identification and quantification of active ingredients remain difficult. Additionally, the mechanisms of action for many TCM components are poorly defined, and their multi-target interactions are inadequately characterized. This mechanistic ambiguity impairs the elucidation of their pharmacokinetic and pharmacodynamic profiles in clinical contexts. Regulatory barriers further complicate clinical translation, as there is a lack of harmonized approval pathways for botanical drugs. Together, these factors limit the widespread adoption of TCMs in CRC therapy. Overcoming these challenges will require advances in analytical methodologies, multi-omics integration, and more adaptive regulatory frameworks to fully harness the therapeutic potential of TCMs in CRC management.

The interplay between tumor metabolism and immune regulation is pivotal in cancer progression and therapeutic response. Lactate, a key byproduct of aerobic glycolysis, plays a central role in establishing an immunosuppressive tumor microenvironment. Beyond acidifying the extracellular milieu—thereby impairing T-cell function—lactate also acts as a signaling molecule, promoting angiogenesis and enhancing the immunosuppressive activities of MDSCs and Tregs (155).

Glucose metabolism is equally critical for T-cell activation. Effector T cells depend on glucose uptake via GLUT1 to sustain their functions. However, glucose competition within the tumor microenvironment—driven by the Warburg effect in cancer cells—can severely restrict T-cell activity (156). Metabolic reprogramming further supports the immunosuppressive functions of MDSCs and Tregs, with MDSCs exhibiting elevated arginine metabolism and reactive oxygen species (ROS) production (157), while Tregs preferentially utilize fatty acid oxidation and specific amino acids (158). Notably, tumor metabolic activity has been linked to PD-L1 expression (159), suggesting that metabolic interventions could enhance the efficacy of immune checkpoint blockade. Early clinical trials are currently evaluating combinations of metabolic inhibitors—such as CA-170, YPD-30, MAX-10181, GS-4224, and BMS-986189 (160)—with immune checkpoint inhibitors across multiple cancer types, including lung cancer, melanoma, and CRC (161). While these approaches show promise, further studies are necessary to optimize therapeutic efficacy and mitigate adverse effects.
